# Genome sequence of type strain of *Staphylococcus aureus* subsp. *aureus*

**DOI:** 10.1186/1757-4749-6-6

**Published:** 2014-03-17

**Authors:** Bong-Soo Kim, Hana Yi, Jongsik Chun, Chang-Jun Cha

**Affiliations:** 1 ChunLab, Inc., Seoul, Republic of Korea; 2 Division of Biosystems Biomedical Science, College of Health Science, Korea University, Seoul, Republic of Korea; 3 Department of Public Health Science, Graduate School, Korea University, Seoul, Republic of Korea; 4 Korea University Guro Hospital, Korea University, Seoul, Republic of Korea; 5 School of Biological Sciences and Bioinformatics Institute, BIO-MAX, Seoul National University, Seoul, Republic of Korea; 6 Department of Systems Biotechnology, Chung-Ang University, Anseong, Republic of Korea

**Keywords:** *Staphylococcus aureus* subsp. *aureus*, Genome sequencing, Type strain, Hybrid assembly

## Abstract

**Background:**

*Staphylococcus aureus* is a pathogen that causes food poisoning and community-associated infection with antibiotic resistance. This species is an indigenous intestinal microbe found in infants and not found in adult intestine. The relatively small genome size and rapid evolution of antibiotic resistance genes in the species have been drawing an increasing attention in public health. To extend our understanding of the species and use the genome data for comparative genomic studies, we sequenced the type strain of *S. aureus* subsp. *aureus* DSM 20231^T^.

**Results:**

Seventeen contigs were generated using hybrid assembly of sequences derived from the Roche 454 and Illumina systems. The length of the genome sequence was 2,902,619 bases with a G + C content of 32.8%. Among the 2,550 annotated CDSs, 44 CDSs were annotated to antibiotic resistance genes and 13 CDSs were related to methicillin resistance. It is interesting to note that this strain was first isolated in 1884 before methicillin was generally used on patients.

**Conclusions:**

The present study analyzed the genome sequence of *S. aureus* subsp. *aureus* type strain as the reference sequence for comparative genomic analyses of clinical isolates. Methicillin resistance genes found in the genome indicate the presence of antibiotic resistance mechanism prior to the usage of antibiotics. Further comparative genomic studies of methicillin-resistant strains based on this reference genome would help to understand the evolution of resistance mechanism and dissemination of resistance genes.

## Background

*Staphylococcus aureus* is a member of normal microbiota in human body and also known as an opportunistic pathogen. This species can cause a broad range of nosocomial and community-associated infections, and the antibiotic resistance of the species has been studied for many years [1]. *S aureus* was also reported as the predominant species in infant feces, and decreased toward adulthood due to the colonization of complex gut microbiota [2,3]. The species can spread through skin-to-skin contact with colonized individuals, and cause a global epidemic as antibiotic resistant strains [4]. Foodborne illness can be caused by enterotoxin-producing *S. aureus* with symptoms such as diarrhea, nausea and abdominal cramps [5]. Recently, *S. aureus* was detected in irritable bowel syndrome (IBS) subjects [6].

Many strains of *S. aureus* subsp*. aureus* were genome-sequenced and submitted to public databases due to the importance in antibiotic resistance and the possibility of nosocomial infections even in health care and community settings [7-9]. However, type strain of this species has not been genome-sequenced yet. Type strain is usually the firstly isolated strain of the species, and exhibits all of the relevant phenotypic and genotypic properties cited in the species circumscriptions. Therefore, the genome sequence of type strain is important to analyze the phenotypic and genotypic characteristics of species. In the present study, we analyzed the whole genome sequence of *S. aureus* subsp*. aureus* type strain as the standard reference genome required for *S. aureus* studies.

## Methods

### Strain information

Type strain of *S. aureus* subsp. *aureus* (DSM 20231^T^) was obtained from Deutsche Sammlung von Mikrooganismen und Zellkulturen GmbH (DSMZ; Barunschweig, Germany). The strain was known to be non-motile, non-spore-forming, Gram-positive cocci (0.5-1.0 μm in diameter), facultatively anaerobic and producing enterotoxin. Optimal growth is observed at 30-37°C on trypticase soy yeast extract media containing 10% NaCl [10].

### Genomic DNA extraction

Genomic DNA was extracted using a Wizard Genomic DNA Isolation kit (Promega, Madison, WI, USA). The concentration of extracted DNA was quantified using a PicoGreen dsDNA Assay kit (Invitrogen, Carlsbad, CA, USA), and the contamination of DNA or cultured strain was checked by sequencing the 16S rRNA gene using the ABI 3730 DNA sequencing machine (Applied Biosystems, Foster City, CA, USA).

### Whole genome sequencing

The draft genome sequence of strain DSM 20231^T^ was determined by a combination of Illumina Genome Analyzer IIx (150 bp paired end) and Roche 454 (8-kb insert paired end) sequencing systems. The sequencing library was prepared with the TruSeq DNA LT Sample Prep kit (Illumina, San Diego, CA, USA) for the Illumina system, and the library for the Roche 454 system was prepared using the GS FLX Titanium Rapid Library Preparation kit (Roche Diagnostics, Branford, CT, USA).

### Assembly and annotation of genome sequence

Sequencing reads obtained from the Illumina system were assembled using the CLC genomic workbench 5.5 (CLC Bio, Denmark), and the reads obtained from the Roche 454 sequencing system were assembled using the GS Assembler 2.6 (Roche Diagnostics). The assembled contigs from each sequencing system were corrected in their order using the published reference genomes. Hybrid assembly of contigs generated by both systems was conducted using the CodonCode Aligner (CodonCode Co. MA, USA). In brief, the contigs generated by each sequencing system were reassembled together using the CodonCode Aligner. Reassembly of hybrid contigs and unassembled contigs were repeated until the number of hybrid contigs did not change. Contigs of short length (<500 bp) were removed from the hybrid result file. Prediction of genes was performed using Glimmer 3 [11], and annotation was conducted by homology search against the Clusters of Orthologous Groups (COG) and SEED database [12,13]. Prediction of multilocus sequencing typing (MLST) was performed using assembled contigs [14].

### Comparative genomics

A total of 441 genome sequences of strains that belong to *S. aureus* subsp. *aureus* were obtained from EzGenome database (http://ezgenome.ezbiocloud.net), and used to calculate average nucleotide identity (ANI) values [15] with strain DSM 20231^T^. For ANI calculation, the query genome was cut into small fragments (1020 bp), and high-scoring pairs between two genome sequences were selected by BLAST algorithm [16]. Then, a dendrogram was constructed using calculated ANI values by the unweighted pair group method.

Five genome sequences (an ANI value of > 99.78% with strain DSM 20231^T^) were selected as the closest genomes and compared with strain DSM 20231^T^ by using comparative genomic method as described previously [17]. Briefly, homologous regions in a target genome to query ORFs were determined using the BLASTN program, and aligned using a pairwise global alignment. The matched region in the subject contig was extracted and saved as a homolog.

## Quality assurance

A potential contamination was evaluated by identification of 16S rRNA gene amplified from extracted DNA before the whole genome sequencing and by comparison of 16S rRNA gene in draft genome after sequencing. 16S rRNA genes in assembled contigs were found using the rRNA selector [18] and identified using the EzTaxon-e database [19]. Bioinformatic assembly was checked by a comparison of the obtained genome sequence with published genomes of the same species using ANI values [15].

## Initial findings

**Figure 1:**
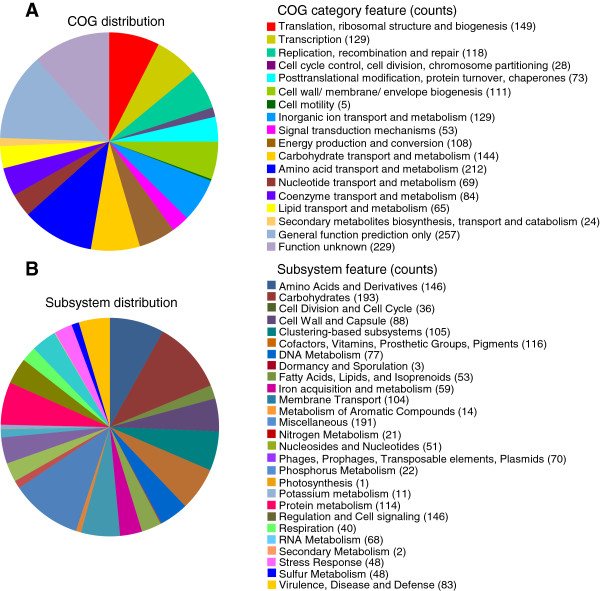
**Statistics of annotated genes for *Staphylococcus aureus *** **subsp. *aureus *** **DSM 20231**^**T **^**based on COG (A) and SEED (B) databases.**

A total of 17 contigs (N50 = 313,118 bp) were generated from a hybrid assembly of reads from Illumina (6,413,077 reads of 150 bp paired end; > 350-fold coverage) and Roche 454 (240,863 reads of 8Kb-insert paired end; > 14-fold coverage) systems. The genome size of strain DSM 20231^T^ was 2,761,522 bases with 32.8% G + C content. The genome contained 2,550 predicted protein-coding sequences (CDSs), 57 tRNA genes and 12 rRNA genes. Results of the genome annotation are shown in Figure 1. For the COG distribution, R (General function prediction only; 257 ORFs), S (Function unknown; 211 ORFs), and E (Amino acid transport and metabolism; 212 ORFs) were abundant categories (over 10% of total COG matched counts). Genes responsible for carbohydrates (193 ORFs), miscellaneous (191 ORFs), amino acid metabolism (146 ORFs) and cell signaling (146 ORFs) were abundant among the SEED subsystem categories.

**Figure 2:**
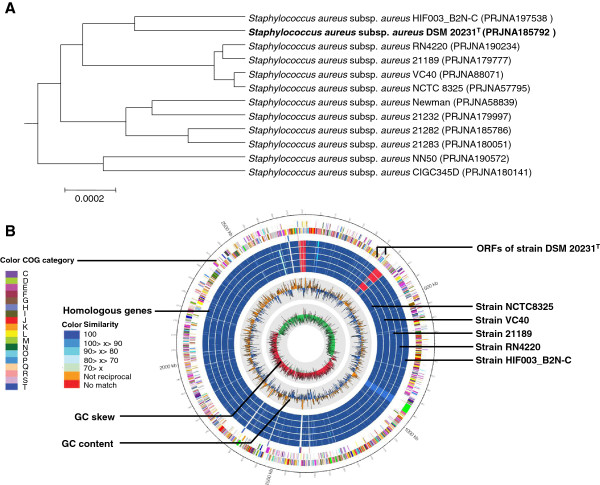
**Genomic relationship of strain DSM 20231**^**T **^**and related *S. aureus *strains. (A)** Genome tree based on ANI values. **(B)** Comparison of homologous genes among five selected genomes in circular representation. The description of each circle was indicated by each line.

The genome tree of *S. aureus* strains was constructed by using ANI calculation (Figure 2A), and strains HIF003-B2N-C, RN4220, 21189, VC40, and NCTC 8325 were chosen based on ANI values for the comparative analysis. Strain HIF003_B2N-C was recovered as the closest genome of the sequenced genome in the genome tree. The number of different gene contents between strains of DSM 20231^T^ and HIF003-B2N-C was 35 ORFs, and the highest different number between them was observed in K (Transcription) and L (Replication, recombination and repair) categories. Genome sequences among selected strains for comparison were similar to each other, and most of the different ORFs were hypothetical proteins, replication-associated proteins, and transposases. Comparison of homologous genes among the selected genomes is given in Figure 2B.

In subsystem distribution of the sequenced genome, 83 genes (4.6% of total subsystem counts) were annotated to virulence, disease and defense category, and 91.6% of genes (76 ORFs) in this category were annotated to be responsible for adhesion and antibiotic resistance. Adhesion to human intestinal mucus and antibiotic resistance of *S. aureus* are important characteristics of pathogens. The highest number of predicted protein sequences (13 CDSs) among 44 CDSs in antibiotic resistance subcategory was annotated to methicillin resistance-related genes (Table 1). In the case of the five related genomes, the numbers of these genes were much smaller than that in the type strain (3 CDSs in HIF003_B2N-C and RN4220, 2 CDSs in NCTC 8325, 1 CDS in VC40, and no hit in strain 21189). The methicillin resistance of *S. aureus* was first reported in 1961 [20]. However, strain DSM 20231^T^ was isolated in 1884 from human pleural fluid [21]. This implies that *S. aureus* had already possessed potential genes for methicillin resistance before methicillin was introduced in 1960. Our finding can provide an insight into history and evolution of methicillin resistance. The predicted MLST of strain DSM 20231^T^ and the five selected strains for comparison were all ST8 in the clonal complex (CC) 8, where the first MRSA clinical isolate is ST250 [1]. In addition, genes related to arsenic resistance, fluoroquinolone resistance, fosfomycin resistance, vancomycin resistance, multidrug resistance efflux pumps, cobalt-zinc-cadmium resistance, and copper homeostasis were annotated in the genome sequence of strain DSM 20231^T^. The presence of antibiotic resistance genes in the genome of this strain implies that antibiotic resistances of this species have evolved for a long time before synthetic antibiotics were used.

**Table 1:** Summary of CDSs annotated to methicillin resistance

**Contig number**	**Length (bp)**	**Seed subsystem**	**Seed function**
2	1,194	Methicillin_resistance_in_*Staphylococci*	FmtA protein involved in methicillin resistance
2	1,245	Methicillin_resistance_in_*Staphylococci*	FmhC protein of FemAB family
2	369	Methicillin_resistance_in_*Staphylococci*	FemC factor involved in methicillin resistance
2	2,523	Methicillin_resistance_in_*Staphylococci*	FmtC (MrpF) protein involved in methicillin resistance
2	1,263	Methicillin_resistance_in_*Staphylococci*	FmhA protein of FemAB family
2	1,260	Methicillin_resistance_in_*Staphylococci*	FmhA protein of FemAB family
3	876	Methicillin_resistance_in_*Staphylococci*	LytH protein involved in methicillin resistance
6	465	Methicillin_resistance_in_*Staphylococci*	HTH domain protein SA1665, binds to mecA promoter region
9	1,185	Methicillin_resistance_in_*Staphylococci*	HmrA protein involved in methicillin resistance
9	7,437	Methicillin_resistance_in_*Staphylococci*	FmtB (Mrp) protein involved in methicillin resistance and cell wall biosynthesis
11	1,266	Methicillin_resistance_in_*Staphylococci*	tRNA-dependent lipid II-glycine ligase (FmhB)
12	1,251	Methicillin_resistance_in_*Staphylococci*	FmhA protein of FemAB family
16	675	Methicillin_resistance_in_*Staphylococci*	Transposase for insertion sequence-like element IS431mec

## Future directions

The genome sequence of *S. aureus* subsp. *aureus* type strain can be used as a standard reference genome sequence for studying *S. aureus* strains including MRSA. Further comparative genome analyses of *S. aureus* strains will provide differences in the genomic contents found in this species and evolutionary information on resistance developments via horizontal gene transfer and mutation. These studies will also help to understand the pathogenesis of the staphylococcal diseases for infection preventions.

## Availability of supporting data

The draft genome sequence of *Staphylococcus aureus* subsp. *aureus* DSM 20231^T^ was deposited at DDBJ/EMBL/GenBank under the accession AMYL00000000. The version described in this paper is the first version AMYL01000000.

## Competing interests

The authors declare that they have no competing interests.

## Authors’ contributions

JC and CC designed the study. BK and HY performed experiments. BK, HY and JC analyzed the sequencing data. BK, HY and CC contributed to the writing of manuscript. All authors read and approved the final manuscript.
